# Established breast cancer risk factors by clinically important tumour characteristics

**DOI:** 10.1038/sj.bjc.6603207

**Published:** 2006-06-06

**Authors:** M García-Closas, L A Brinton, J Lissowska, N Chatterjee, B Peplonska, W F Anderson, N Szeszenia-Dąbrowska, A Bardin-Mikolajczak, W Zatonski, A Blair, Z Kalaylioglu, G Rymkiewicz, D Mazepa-Sikora, R Kordek, S Lukaszek, M E Sherman

**Affiliations:** 1 Division of Cancer Epidemiology and Genetics, National Cancer Institute, National Institute of Health, 6120 Executive Blvd. Room 7076, Rockville, MD 20852-7234, USA; 2 Department of Cancer Epidemiology and Prevention, Cancer Center and M Sklodowska-Curie Institute of Oncology, Warsaw, Poland; 3 Nofer Institute of Occupational Medicine, Łódź, Poland; 4 IMS, Silver Spring, MD, USA; 5 Department of Pathology, Cancer Center and M Sklodowska-Curie Institute of Oncology, Warsaw, Poland; 6 Department of Pathology, Medical University of Łódź, Łódź, Poland; 7 Department of Clinical Pathomorphology, Polish Mother's Memorial Hospital-Research Institute, Łódź, Poland

**Keywords:** breast cancer, epidemiology, aetiologic heterogeneity, histology

## Abstract

Breast cancer is a morphologically and clinically heterogeneous disease; however, it is less clear how risk factors relate to tumour features. We evaluated risk factors by tumour characteristics (histopathologic type, grade, size, and nodal status) in a population-based case–control of 2386 breast cancers and 2502 controls in Poland. Use of a novel extension of the polytomous logistic regression permitted simultaneous modelling of multiple tumour characteristics. Late age at first full-term birth was associated with increased risk of large (>2 cm) tumours (odds ratios (95% confidence intervals) 1.19 (1.07–1.33) for a 5-year increase in age), but not smaller tumours (*P* for heterogeneity adjusting for other tumour features (*P*_het_)=0.007). On the other hand, multiparity was associated with reduced risk for small tumours (0.76 (0.68–0.86) per additional birth; *P*_het_=0.004). Consideration of all tumour characteristics simultaneously revealed that current or recent use of combined hormone replacement therapy was associated with risk of small (2.29 (1.66–3.15)) and grade 1 (3.36 (2.22–5.08)) tumours (*P*_het_=0.05 for size and 0.0008 for grade 1 *vs* 3), rather than specific histopathologic types (*P*_het_=0.63 for ductal *vs* lobular). Finally, elevated body mass index was associated with larger tumour size among both pre- and postmenopausal women (*P*_het_=0.05 and 0.0001, respectively). None of these relationships were explained by hormone receptor status of the tumours. In conclusion, these data support distinctive risk factor relationships by tumour characteristics of prognostic relevance. These findings might be useful in developing targeted prevention efforts.

Breast cancers vary greatly in clinical behaviour, histopathologic appearance, and molecular alterations. In addition, age-specific incidence rates for breast cancer vary by histologic tumour type (Anderson *et al*, 2004a), stage, and grade (Anderson *et al*, 2005) and hormone receptor status (Yasui and Potter, 1999; Anderson *et al*, 2002), suggesting that breast cancers might be aetiologically distinct. Thus, demonstrating that specific epidemiologic risk factors differ by clinically important tumour characteristics may facilitate the development of targeted prevention efforts. However, most epidemiologic studies performed to date have treated breast cancer as a single disease with a common set of risk predictors.

Mounting albeit still limited evidence from epidemiological studies suggests that breast cancer predictors vary by histological type and hormone receptor status. Specifically, combined oestrogen and progestin hormone replacement therapy (HRT) (Li *et al*, 2000; Chen *et al*, 2002; Daling *et al*, 2002; Newcomb *et al*, 2002; Li *et al*, 2003; Newcomer *et al*, 2003), and possibly late age at first birth (LiVolsi *et al*, 1982; Ewertz and Duffy, 1988; Stalsberg *et al*, 1989) may be more strongly associated with lobular as compared to ductal carcinomas. Epidemiologic data also suggest that hormone-related risk factors vary by hormone receptor status (Althuis *et al*, 2004).

In this report, we evaluated heterogeneity of established breast cancer risk factors stratified by histopathological type, tumour grade, size and nodal status, in a large population-based case–control study in Poland. We used a novel statistical method to disentangle the independent effects of these correlated tumour features, as well as to adjust for hormone receptor status (Chatterjee, 2004).

## MATERIALS AND METHODS

### Study population

A population-based breast cancer case–control study was conducted in Poland between 2000 and 2003. Eligible cases were women residing in Warsaw or Łódź, 20–74 years of age, and recently diagnosed with either histologically or cytologically confirmed incident *in situ* or invasive breast cancer. Cases were recruited through a rapid identification system organized at five participating hospitals, which identified about 90% of eligible cases, and cancer registries. Eligible control subjects were residents of Warsaw and Łódź without a history of breast cancer at enrollment. The Polish Electronic System, a database with demographic information from all residents of Poland, was used to randomly select controls frequency matched to cases on city and age in 5-year categories.

A total of 2386 cases (79% of eligibles) and 2502 controls (69% of eligibles) agreed to participate in a personal interview regarding known and suspected risk factors for breast cancer. The main reasons of nonparticipation for cases and controls, respectively, were refusal (19 and 25%) and unable to locate (2 and 6%). Interviews were conducted a median of 6.8 weeks following diagnosis for cases, and 2.4 weeks following identification for controls. A signed informed consent to participate in the study was obtained from all participants in accordance with the National Cancer Institute and local Institutional Review Boards.

### Risk factor information

Women were considered premenopausal if they reported having natural menstrual periods at the time of interview, postmenopausal if periods had stopped, and unclear menopausal status if HRT use had been started before the natural periods stopped. Women who reported having breastfed for 1 month or less were considered as having never breastfed. Women who had used oral contraceptives or oral HRT for 1 month or less were classified as non-users. Users of oral HRT were further classified as current or recent users (<2 years since last use) of combined (estrogen and progesterone) HRT, past users (⩾2 years) of combined HRT, and users of oestrogen or progesterone HRT alone. Body mass index (BMI) was calculated using current weight (kg) divided by standing height (m) squared as measured by a trained nurse. For 114 cases and 156 controls without measures of weight or height, BMI was calculated using self-reported information. Women were classified as non-drinkers if they reported having consumed 12 or fewer alcoholic drinks in their lifetime or they reported consuming less than one drink per month for 6 months without ever having had more than five drinks on any one occasion. Women were considered as having a history of benign breast disease if they reported having had a benign breast biopsy 1 year prior to either the diagnosis date (for cases) or the date of interview (for controls).

### Pathology information

Information about diagnostic and treatment procedures was obtained from the medical records, and surgical pathology forms that were completed after clinical sign-out of cases. The surgical pathology form documented macroscopic (type and size of surgical specimens and location and size of masses) and microscopic (histopathologic diagnosis, grade, and status of axillary and other lymph nodes) features. Results for oestrogen receptor (ER) and progesterone receptor (PR) assays performed in Poland were obtained from medical records. In 91% of cases with receptor status information, assays were performed using immunohistochemistry, with biochemical methods used in the remainder.

A single US pathologist (MES) reviewed haematoxylin and eosin-stained slides to confirm case status and provide uniform histologic classification. Final diagnoses for 1958 (82%) cases with tumour slides available were based on the pathology review by MES. Tumours were classified as ductal not otherwise specified (NOS) or lobular if they demonstrated a predominant histopathologic appearance; tumours containing mixed patterns were designated as mixed carcinomas. Carcinomas were classified as tubular or cribriform if the characteristic well-differentiated patterns together accounted for 90% of the tumour area. Tubular carcinomas and ductal carcinomas, NOS, grade 1 share morphologic and clinical features. Accordingly, we combined these types in some analyses. Studies have also suggested that low-grade ductal or tubular carcinomas are related to infiltrative lobular carcinomas, with the former being the most highly differentiated and the later the most undifferentiated extreme, a view that is supported by the observation of patterns of low-grade ductal and lobular carcinomas together in tubulo-lobular carcinomas (Fisher *et al*, 1977; Eusebi *et al*, 1979; Green *et al*, 1997). Thus, all of these types have also been combined in some analyses. Grading was performed according to Elston criteria (Elston and Ellis, 1998), with the modification that mitotic rate was estimated.

For the remaining 428 cases without slides available for review, the diagnosis in Poland was considered the final diagnosis. The percent agreement between MES and Poland for invasive diagnosis was 80% for ductal NOS, 68% for lobular, and 18% for mixed carcinomas. The disagreement was mainly explained by reclassification of mixed type tumours as ductals or lobulars.

### Statistical analysis

Logistic regression was used to estimate adjusted odds ratios (ORs) and associated 95% confidence intervals (CI) from models that included all risk factors simultaneously. Models included continuous terms for age at menarche, number of full-term births, age at first full-term birth, age at menopause and BMI, and dummy variables for education levels, nulliparity, oral HRT use (never user, current or recent use of combined HRT, past use of combined HRT, use of other HRT), family history, history of a benign biopsy, ever had a screening mammography and menopausal status (premenopausal, postmenopausal, and unclear), current age in 5-year categories and study site. Because the association between BMI and breast cancer risk is known to differ by menopausal status, our models included separate terms for pre-and postmenopausal women. Estimation of ORs for different categories of variables considered as continuous indicated that the log-linear assumption was reasonable. Standard polytomous logistic regression was used to estimate ORs and 95% CI for different tumour types. Heterogeneity between risk factor ORs for different tumour types was assessed using logistic regression analyses restricted to cases (case-only analyses). An extension of the polytomous logistic regression model was used to evaluate heterogeneity in risk factor ORs by multiple tumour characteristics simultaneously (Chatterjee, 2004). This method allowed us to evaluate which of several correlated tumour features, that is, histopathologic type, grade, size and nodal status, was most important in determining risk factor associations. Oestrogen receptor and PR status were also included as potential confounders. These analyses included cases diagnosed with major histological subtypes (ductal carcinomas, NOS; tubular carcinomas (classified as grade 1 ductal carcinoma, NOS); lobular; and mixed carcinomas; total *N*=1964). Odds ratios (95% CI) and corresponding *P*-values (*P*_het_) reported from these analyses measure the association between tumour characteristics and risk factors, similar to case-only analyses.

## RESULTS

### Characteristics of study population

About two-thirds of women were recruited in Warsaw and one-third in Łódź, with a mean age (±s.d.) of 56 (±10) years. Distribution of characteristics for cases and controls were consistent with most established risk factors (Table 1). Use of oral contraceptives or HRT, alcohol consumption, and mammographic screening were relatively uncommon in this population.

Approximately 94% of all cases in the study had a tumour with an invasive component, with ductal carcinomas, NOS, accounting for 58% of invasive cases, lobular carcinoma for 16%, and mixed carcinoma for 12% (Table 2). Lobular and mixed carcinomas were better differentiated than ductal carcinomas, NOS, whereas the distributions of tumour size and axillary lymph node metastases were similar across these tumour types (Table 3). Among ductal carcinomas, NOS, 59% were ER positive and 50% were PR positive; percentages for receptor detection were higher among lobular, mixed, tubular, and tubulo-lobular carcinomas. Analyses for heterogeneity between risk factors in the remainder of the manuscript was restricted to tumours with known invasive component (*N*=2144).

### Predictors of invasive breast cancer risk by tumour histology

Overall, breast cancer risk was directly associated with higher level of education, late age at first full-term birth, late age at menopause, current or recent use of combined HRT, family history of breast cancer and prior benign breast biopsy (Supplementary Table 4 online). On the other hand, breast cancer risk was inversely associated with late age at menarche, multiparity, and high BMI in pre-menopausal women (Supplementary Table 4 online). Among postmenopausal women, BMI was not associated with overall breast cancer risk. Oral contraceptive use and alcohol consumption were uncommon and unrelated to overall risk (data not shown); these factors were therefore not further considered.

**Figure 1:**
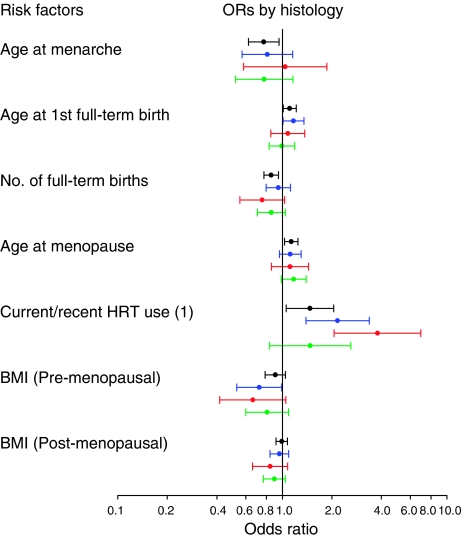
Predictors of invasive breast cancer risk in the Polish Breast Cancer Study by histological subtypes. Odds ratios (95% CI) for ductal carcinomas, NOS (*N*=1,251) are shown in black, for lobular carcinomas (*N*=342) in blue, for tubular carcinomas (*N*=119) in red, and for mixed carcinoma (*N*=252) in green. Numbers in brackets denote statistically significant heterogeneity of ORs for lobular, tubular, and mixed compared to ductal carcinomas, NOS, respectively, based on standard polytomous logistic regression among cases: (1) 0.13, 0.002, and 0.98. Analyses are adjusted for age, study site, menopausal status, education level, family history, prior benign breast biopsy, screening mammogram, and all other factors shown in the figure. Comparison groups are 5-year increases for ages at menarche, first full-term birth, and menopause; each additional birth for number of full-term births; never HRT users for current or recent use of combined HRT; 5 unit increases for BMI.

Most predictors of risk were similar across histologic types, with the exception of current or recent use of combined HRT, which was associated with a greater risk for lobular and tubular as compared to ductal carcinomas, NOS (Figure 1; Supplementary Table 4 online). Current or recent use of combined HRT was also associated with an increased risk of tubulo-lobular carcinoma (*N*=50; ORs (95% CI) of 1.85 (0.67–5.09)), although the precision of the estimate was limited by small numbers. Risk factor associations were similar for mixed and ductal carcinomas, NOS (Figure 1; Supplementary Table 4 online).

### Invasive breast cancer risk by tumour grade, size, and nodal status

Differences in risk factors by tumour grade, size, and nodal status were evaluated for the major histological types (ductal NOS, tubular, lobular, and mixed tumours). For these analyses, tubular tumours were included with ductal carcinomas, NOS, grade 1 since they are a grade 1 ductal variant with similar morphological and clinical features.

**Figure 2:**
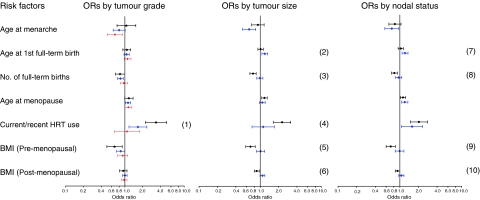
Predictors of invasive breast cancer (ductal carcinomas, NOS, tubular, lobular, and mixed types) in the Polish Breast Cancer Study by tumour grade, size, and nodal status. Odds ratio (95% CI) for grade 1 (*N*=333), small (⩽2 cm, *N*=988), or node negative (*N*=1084) tumours are shown in black; for grade 2 (*N*=979), large (>2 cm, *N*=796), or node positive (*N*=708) are shown in blue; and for grade 3 (*N*=500) are shown in red. Numbers in brackets denote statistically significant heterogeneity of ORs based on standard polytomous logistic regression among cases: (1) 0.001 and 0.00008 for grades 2 and 3 compared to grade 1 tumours, respectively; (2) 0.02, (3) 0.0019, (4) 0.001, (5) 0.0006, and (6) 0.0005 for small (⩽2 cm) compared to large (>2 cm) tumours; and (7) 0.02, (8) 0.006, (9) 0.002, and (10) 0.02 for node positive compared to node negative tumours. Analyses are adjusted for age, study site, menopausal status, education level, family history, prior benign breast biopsy, screening mammogram, and all other factors shown in the figure. Comparison groups are 5-year increases for ages at menarche, first full-term birth, menopause; each additional birth for number of full-term births; never HRT users for current or recent use of combined HRT; 5 unit increases for BMI.

Delayed age at first full-term birth was associated with increased risk for tumours that were large (>2 cm) (OR (95% CI)=1.19 (1.07–1.33)) or with positive nodes (1.12 (1.08–1.35)). In contrast, the reduced breast cancer risk associated with multiparity was strongest for carcinomas that were small (⩽2 cm) (0.76 (0.68–0.86)) or node negative (0.82 (0.73–0.91)) (Figure 2; Supplementary Table 6 online). Increased breast cancer risk associated with current or recent use of combined HRT was limited to low-grade carcinomas (3.36 (2.22–5.08)) and tumours of small size (2.29 (1.66–315)) (Figure 2; Supplementary Tables 5 and 6 online). For the combined group including grade 1 ductal NOS/tubular, lobular, and tubulo-lobular carcinomas (*N*=628), HRT use was associated with an OR (95% CI) of 2.77 (1.96–3.91).

Elevated BMI in pre-menopausal women was associated with reduced risks for tumours that were small or node negative. Elevated BMI in postmenopausal women was not associated with overall breast cancer risk; however, data suggested an association with decreased risk of small or node negative tumours, and a small increased risk of larger or node positive tumours (Figure 2; Supplementary Table 6 online). Additional risk factor data are shown as supplementary data (Supplementary Tables 5 and 6 online).

### Simultaneous analysis of tumour characteristics

In this section, we evaluate the association between predictors of breast cancer risk and different tumour characteristics simultaneously using a novel extension of polytomous logistic regression to account for multiple disease outcomes (Chatterjee, 2004). Odds ratios (95% CI) from these analyses and their corresponding *P*-values (*P*_het_; shown in Supplementary Tables 5 and 6 online) measure the association between tumour characteristics and risk factors, similar to case-only analyses.

Late age at first full-term birth and multiparity were associated with larger tumour size: OR (95% CI) for size >2 cm *vs* ⩽2 cm=1.23 (1.06–1.44), *P*_het_=0.007 for 5-year increase in age at first birth; and 1.30 (1.09–1.55), *P*_het_=0.004 for each additional full-term birth. However, associations between these risk factors and nodal status found in the standard polytomous logistic regression models were no longer significant in models considering multiple tumour characteristics simultaneously (Supplementary Tables 5 and 6 online).

Recent or current use of combined HRT was significantly related to low tumour grade (0.38 (0.23–0.65), *P*_het_=0.0003, and 0.29 (0.14–0.60), *P*_het_=0.0008 for grades 2 and 3 compared to grade 1, respectively), and smaller tumour size (0.60 (0.36–0.99), *P*_het_=0.05 for tumours >2 cm *vs* ⩽2 cm), but the association with lobular type suggested in the standard logisitic analyses (Figure 1) was no longer significant (1.14 (0.66–2.00), *P*_het_=0.63 for lobular *vs* ductal tumours). This model indicated that HRT use is related to tumour grade, independent of the histological type. Indeed, stratification of lobular tumours by grade 1 (*N*=60) and grades 2 or 3 (*N*=250) indicated a stronger increase in breast cancer risk for grade 1 (3.62 (1.52–8.63)) *vs* grades 2 or 3 (2.18 (1.30–3.65)), *P*_het_=0.12.

Elevated BMI in pre- and postmenopausal women was associated with larger tumour size (1.28 (1.00–1.64), *P*_het_=0.05; 1.30 (1.14–1.49), *P*_het_=0.0001, respectively); however, the association with nodal status was present for pre-menopausal women (1.28 (1.00–1.62) *P*_het_=0.04) but not for postmenopausal women (1.04 (0.92–1.19), *P*_het_=0.51).

## DISCUSSION

Analysis of data from this large population-based case–control study provides convincing evidence that breast cancer risk factors differ by clinically important tumour features, including histopathological type, grade, size, and nodal status. Thus, exposures that influence the risk of developing breast cancer might also affect the biology and clinical behaviour of the tumours that arise. These findings parallel data suggesting that molecular profiles of breast cancers are generally fixed at inception and represent important determinants of clinical behavior (Lacroix *et al*, 2004). Accordingly, understanding relationships between risk factors for breast cancer and tumour characteristics could have implications for screening and prevention.

Similar to a previous case–control study (Wohlfahrt *et al*, 1999), we found that delayed age at first full-term birth was associated with increased risk of tumours that were large or node positive, whereas multiparity was associated with reduced risk for small tumours. Furthermore, analyses using a novel statistical method, which considered all tumour characteristics simultaneously, indicated that late age at first full-term birth and multiparity were more strongly related to larger tumour size than nodal invasion. Thus, these reproductive factors might act primarily to enhance tumour growth rate or delay detection. Either explanation would favour implementing improved prevention efforts for these women. Although the mechanisms responsible for the associations of delayed age at first birth with poor prognostic features are unknown, continuous exposure to cyclic hormones uninterrupted by the dramatic differentiation and remodelling effects of pregnancy on breast tissue might play an important role. In contrast to some previous reports (LiVolsi *et al*, 1982; Ewertz and Duffy, 1988; Stalsberg *et al*, 1989), we and others (Morrison, 1976; Wohlfahrt *et al*, 1999) did not find that late age at first full-term birth was more strongly associated with lobular as compared with ductal carcinoma, NOS.

As previously reported, we found that HRT use was more strongly related to lobular (Gapstur *et al*, 1999; Li *et al*, 2000; Chen *et al*, 2002; Daling *et al*, 2002; Newcomb *et al*, 2002; Li *et al*, 2003; Newcomer *et al*, 2003) and tubular (Manjer *et al*, 2001) carcinomas than to other histopathologic types. In addition, we observed a stronger association between HRT use and risk for grade 1 ductal carcinomas, NOS, and tubulo-lobular carcinomas. This observation is consistent with the hypothesis that low-grade ductal or tubular and lobular carcinomas are aetiologically related and may represent the morphologic extremes of tumours (with the former being the most highly differentiated and the later the most undifferentiated extremes) that share a common carcinogenic pathway (Fisher *et al*, 1977; Eusebi *et al*, 1979; Green *et al*, 1997).

This report and others have found that combined HRT use is also associated with low grade or small tumours (Collaborative Group on Hormonal Factors in Breast Cancer, 1997; Gapstur *et al*, 1999; Li *et al*, 2000; Manjer *et al*, 2001). Consideration of all tumour characteristics simultaneously in our analyses indicated that HRT use is primarily associated with tumour grade and to a lesser extent, with tumour size, whereas associations with histopathologic type or nodal status were not significant. It is possible that these findings reflect a detection bias associated with increased screening among HRT users; however, we found similar associations among screened and unscreened women (data not shown). In addition, it is known that HRT increases breast density, which decreases the sensitivity of mammography, and that mammography is insensitive in detecting lobular carcinomas. From a public health perspective, it is reassuring that the excess breast cancer risk associated with HRT use is related mainly to tumours with good prognostic features.

Findings from this case–control study provide support for an association between obesity and later stage at diagnosis, as it has been reported in most previous studies, mostly case-series (Daniell, 1988; Ingram *et al*, 1989; Verreault *et al*, 1989; Reeves *et al*, 1996; Jones *et al*, 1997; Hall *et al*, 1999; Cui *et al*, 2002), with a few exceptions (Donegan *et al*, 1978; Howson *et al*, 1986). In addition, consideration of all tumour characteristics simultaneously, suggested that obesity is primarily associated with larger tumour size rather than nodal status, particularly among postmenopausal women. Case–control analyses indicated that the association between obesity and larger tumour size in pre-menopausal women reflects a protection of obesity against small but not larger tumours, as it has been previously reported (Hall *et al*, 1999). This finding could reflect failed detection of smaller tumours by self or medical examination since tumours are more difficult to palpate in obese women. Among postmenopausal women only, high BMI was also associated with a small increase in risk for large tumours, which is consistent with growth enhancement due to higher levels of circulating hormones among obese than non-obese postmenopausal women. Previous studies have suggested that BMI is associated with hormone receptor-positive tumours which could confound the observed association with tumour size (Althuis *et al*, 2004). However, in our data, associations between BMI and tumour size were independent of hormone receptor status.

It has been suggested that tumours with poor prognostic features (i.e. high grade, large size, node positive, ER negative) differ aetiologically (Mueller, 1988; Anderson *et al*, 2004b; Li *et al*, 2005). Our data support this notion, challenging the view that tumour aggressiveness results entirely from stochastic molecular events that occur over time (Hellman and Harris, 2000). It is unclear whether risk factors directly affect prognosis, indirectly affect outcomes by influencing tumour characteristics at presentation or are unrelated to the clinical course.

Strengths of our study include large sample size, high participation rates, and standardised histopathologic assessment by an independent pathology review. In addition, we considered different tumour characteristics simultaneously using a novel statistical method (Chatterjee, 2004) which allowed us to evaluate the independent association of these characteristics, and adjust for hormone receptor status of the tumour. Although this study population had higher percentage of tumours with adverse prognostic features than those observed in other Western populations, most known breast cancer risk factors were present in similar magnitude as previously reported, indicating that our findings should be generalisable to other populations.

In summary, this population-based study provides evidence that breast cancer risk factors are associated with clinically important tumour characteristics, suggesting that aetiological factors may affect the biological behaviour of breast cancers. In addition, these data suggest that postmenopausal women who are nulliparous have later ages at first birth and are obese might benefit from more frequent screening.

## Figures and Tables

**Table 1:** Characteristics of the study population in the Polish Breast Cancer Study

	**Cases**	**Controls**
Study characteristic	*N*=2386	*N*=2502
Age in years (mean±s.d.)	55.8±10.0	55.9±10.1
Study site (% Warsaw)	65	63
Education level (% college degree)	25	15
Marital status (% married)	62	63
Age at menarche in years (mean±s.d.)	13.5±1.7	13.7±1.7
Parity (% parous)	86	89
Number of full-term births (mean±s.d.)	1.7±0.8	1.9±0.8
Age at first full-term birth (mean±s.d.)	24.5±4.6	23.6±4.2
Breastfeeding among women with live births (% ever)	78	81
Oral contraceptive use (% ever)	12	10
Menopausal status (% postmenopausal)	75	70
Type of menopause among postmenopausal women (% natural)	77	84
Age at menopause (mean±s.d.)	49.6±4.6	49.2±5.0
*Use of oral HRT among postmenopausal women (% ever)*		
Never	77	83
Current use of combined therapy (<6 months)	7	4
Recent use of combined therapy (6 months–<2 years)	4	2
Past use of combined therapy (last use 2 or more years ago)	4	5
Ever used *E* or *P* alone	7	7
Duration of combined HRT among current/recent users (mean±s.d.)	11.1±12.5	9.7±10.7
Current BMI among premenopausal (mean±s.d.)	25.4±4.9	26.4±5.1
Current BMI among postmenopausal (mean±s.d.)	27.9±5.4	28.6±5.4
Alcohol consumption (% ever)	33	32
Family history of breast cancer in first-degree relatives (%)	10	6
Prior benign breast bipospy (%)	10	6
Ever had a screening mammogram (%)	62	54

BMI=body mass index; HRT=hormone replacement therapy.

**Table 2:** Histological types of breast cancer tumours in the Polish Breast Cancer Study (*N*=2386)

	***N***	**%**
*Invasiveness*		
*In situ*	135	6
Invasive component	2144	94
Other	11	0.5
Unknown	96	a
*Tumours with invasive component* b		
Ductal NOS^c^	1251	58
Lobular	341	16
Mixed	252	12
Tubular	119	6
Tubulobular	50	2
Medullary	16	1
Papillary	7	0.3
Mucinous	20	1
Other primary carcinoma	83	4
Other malignant tumour	3	0.1

a Cases with cytological but no histological confirmation.

b Two cases had missing information.

c NOS=not otherwise specified.

**Table 3:** Characteristics of different histological types of invasive breast cancer tumours in the Polish Breast Cancer Study

	**Ductal NOS^a^**		**Lobular**		**Mixed**		**Tubular**		**Tubulo-lobular**	
	***N*=1251**		***N*=342**		***N*=252**		***N*=119**		***N*=50**	
	***N***	**%**	***N***	**%**	***N***	**%**	***N***	**%**	***N***	**%**
*Grade*										
1 (well differentiated)	121	11	60	19	40	17	112	100	33	66
2 (moderately differentiated)	591	51	228	74	160	66	0		17	34
3 (poorly differentiated)	437	38	22	7	41	17	0		0	0
Unknown	102		32		11		7		0	
*Size (cm)*										
T1: ⩽2.0										
T1a: ⩽0.5	13	1	2	1	1	0	11	9	1	2
T1b: >0.5–⩽1.0	116	10	34	11	27	11	37	31	7	14
T1c: >1.0–⩽2.0	466	41	127	41	101	43	53	45	34	68
T2: >2–⩽5	479	43	133	42	99	42	7	6	8	16
T3: >5	52	5	17	5	9	4	11	9	0	0
Unknown	125		29		15		0			
*Number of positive* nodes										
None	677	59	193	62	121	51	93	89	34	68
1-3	285	25	65	21	71	30	9	9	9	18
⩾ 4	177	16	55	18	43	18	3	3	7	14
Unknown	112		29		17		14			
*ER status*										
Negative	398	41	56	20	59	30	10	12	4	10
Positive	576	59	224	80	139	70	76	88	35	90
Unknown	277		62		54		33		11	
*PR status*										
Negative	483	50	107	38	66	33	27	32	7	18
Positive	487	50	173	62	132	67	58	68	32	82
Unknown	281		62		54		34		11	
Age at diagnosis (mean±s.d.)	55.2	±10.4	57.0	±9.3	56.8	±10.0	55.7	±8.1	53.8	±8.6

a NOS=not otherwise specified.
