# Supplementary material for: Established breast cancer risk factors by clinically important tumour characteristics
Source: Br J Cancer. 2006 Jun 6;95(1):123–9. doi: 10.1038/sj.bjc.6603207 (PMC2360503; doi:10.1038/sj.bjc.6603207)
Supplement: Supplementary Table 4 [file 95-6603207x1.doc]

Table 4 (online): Reproductive and hormonal risk factors for invasive breast cancer in the Polish Breast Cancer Study* by histological subtypes

*2,235 cases and 2,482 controls were included in final models after excluding subjects with missing data on age at first full term birth, menopausal status, age at menopause, family history of breast cancer and BMI.

**OR adjusted for age (5 year categories), study site, menopausal status, parity and all the other factors shown in the table.

*** P-values from standard polytomous logistic regression models adjusting for same factors as indicated above, among cases only.
