# Supplementary material for: Established breast cancer risk factors by clinically important tumour characteristics
Source: Br J Cancer. 2006 Jun 6;95(1):123–9. doi: 10.1038/sj.bjc.6603207 (PMC2360503; doi:10.1038/sj.bjc.6603207)
Supplement: Supplementary Table 5 [file 95-6603207x2.doc]

Table 5 (online): Reproductive and hormonal breast cancer risk factors in the Polish Breast Cancer Study for 1,964 invasive cases (ductal NOS, tubular. lobular, mixed types) and 2,502 controls by tumor grade.

*OR adjusted for age (5 year categories), study site, menopausal status, parity and all the other factors shown in the table.

** P-values from standard polytomous logistic regression models among cases only; and from an extension of the polytomous regression models adjusting for tumor size, nodal status, ER and PR status.
