# Supplementary material for: Established breast cancer risk factors by clinically important tumour characteristics
Source: Br J Cancer. 2006 Jun 6;95(1):123–9. doi: 10.1038/sj.bjc.6603207 (PMC2360503; doi:10.1038/sj.bjc.6603207)
Supplement: Supplementary Table 6 [file 95-6603207x3.doc]

Table 6 (online): Reproductive and hormonal breast cancer risk factors in the Polish Breast Cancer Study for 1,964 invasive cases (ductal NOS, tubular. lobular, mixed types) and 2,502 controls by tumor size and nodal status.

*OR adjusted for age (5 year categories), study site, menopausal status, parity and all the other factors shown in the table.

** P-values from standard polytomous logistic regression models among cases only; and from an extension of the polytomous regression models adjusting for tumor grade, size, nodal status, ER and PR status.
